# Connexin43 Suppresses Lung Cancer Stem Cells

**DOI:** 10.3390/cancers11020175

**Published:** 2019-02-02

**Authors:** Randall J. Ruch

**Affiliations:** Department of Cancer Biology, University of Toledo Health Science Center, Toledo, OH 43614, USA; Randall.Ruch@utoledo.edu; Tel.: +1-419-383-4131

**Keywords:** lung cancer, cancer stem cell, gap junctions, connexin, cell communication

## Abstract

Alterations in gap junctions and their protein components, connexins, have been associated with neoplastic transformation and drug resistance, and more recently have been shown to play important roles in cancer stem cells (CSCs). However, there is less knowledge of connexins and gap junctions in lung CSCs. To address this, Connexin43 (Cx43), the major human lung epithelial gap junction protein, was expressed ectopically in poorly expressing National Cancer Institute-125 (NCI-H125) metastatic human lung adenocarcinoma cells, and phenotypic characteristics of malignant cells and abundance of CSCs were evaluated. The ectopic expression of Cx43 resulted in the formation of functional gap junctions; a more epithelial morphology; reduced proliferation, invasion, colony formation, tumorsphere formation, pluripotency marker expression, and percentage of aldehyde dehydrogenase (ALDH)-positive cells; and increased cisplatin sensitivity. Similarly, in NCI-H522 (human lung adenocarcinoma) and NCI-H661 (human lung large cell carcinoma) cell lines, which express Cx43 and functional gap junctions endogenously, the Cx43 content was lower in tumorspheres and ALDH-positive cells than in bulk cells. These results demonstrate that Cx43 can reverse several neoplastic characteristics and reduce the abundance of human lung CSCs.

## 1. Introduction

Cancer stem cells (CSCs) are a population of cells that have high tumorigenic potential, self-renewal capacity, and resistance to therapy [1,2]. The intense study of CSCs over the past two decades has led to a much greater understanding of their biology. However, whether gap junctions and their protein components (connexins) are expressed in CSCs and how this impacts the CSC phenotype, including resistance to treatment, has not been widely investigated.

Gap junctions are intercellular junctions present in all human tissues and consist of plasma membrane-localized clusters of aqueous channels that connect the interiors of adjacent cells. The channels are comprised of proteins known as connexins of which there are 20 human forms. The channels are approximately 1.5 nm in diameter and allow molecules less than approximately 1 kDa in mass (water, amino acids, simple sugars, second messengers, etc.) to diffuse rapidly between cells. Larger molecules such as proteins and nucleic acids are excluded, although some large, linear molecules such as microRNA can diffuse between cells through gap junction channels. This molecular movement, known as gap junctional intercellular communication (GJIC), is important in normal physiological processes such as homeostasis, growth regulation, coordination of cellular responses to stimuli, and apoptosis. In contrast, aberrant connexin expression and defective GJIC are involved in many diseases such as cancer, cardiac arrhythmia, dysfunctional labor, deafness, neurodegeneration, and cataract formation [3,4].

A limited number of studies of GJIC and connexin expression in CSCs have been undertaken with conflicting results in some cases [5,6,7,8,9,10,11]. In glioma cells, connexin43 (Cx43) reversed stem cell abundance and phenotype through a c-Src–dependent mechanism [6] or through modulation of E-cadherin [11]. In contrast, Cx43 was highly reduced and connexin 46 (Cx46) was elevated in glioblastoma stem cells compared to bulk cells [7]. In breast cancer, connexin 26 (Cx26) was elevated in CSCs of triple-negative breast cancer and maintained stemness through interactions with the pluripotency factor Nanog [10]. Ectopic expression of connexin32 (Cx32) increased the number of cancer stem cells in hepatoma [8]. Higher levels of Cx43 were seen in A549 lung CSCs compared to bulk cells [9].

Human and rodent lung cancer cells typically exhibit reduced GJIC due to reduced connexin expression, and ectopic connexin expression can restore cell–cell coupling and a more normal phenotype; however, whether lung CSCs were affected by upregulated connexin expression has not been addressed [12,13,14,15,16,17,18,19,20]. Therefore in the present study, this paper investigates into whether connexin43 (Cx43), the major gap junction protein expressed by lung epithelial cells [21], impacts human non-small cell lung CSCs.

## 2. Results

### 2.1. Stable Transfection of H125 Cells with Cx43

**Figure 1:**
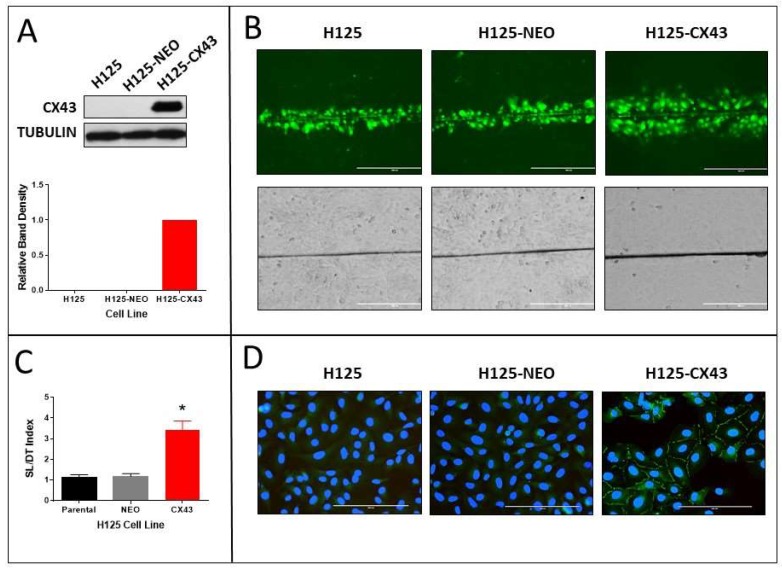
Stable transfection of connexin43 (Cx43) in H125 cells (H125-CX43) increases gap junction formation, gap junctional intercellular communication (GJIC), and E-cadherin and β-catenin localization to the plasma membrane (non-transfected parental cells are designated H125 and empty vector-transfected cells are designated H125-NEO). (**A**) Western blot of Cx43 and densitometric analysis of band densities normalized to tubulin loading control (*n* = 3 replicate experiments); (**B**) scrape-loading/dye-transfer assay for GJIC showing Lucifer Yellow-fluorescent dye-loaded cells (top panels) and bright field images (bottom panels), scale bars: 400 µm; (**C**) quantification of average number of dye-loaded cells perpendicular to the scrape (* *p* < 0.01, Student’s *t*-test, mean ± S.D., *n* = 4 replicate experiments); (**D**) fluorescent fluorescein isothiocyanate (FITC) immunostaining of Cx43 with 4′,6-diamidino-2-phenylindole (DAPI) staining of nuclei, scale bars: 200 µm.

H125 cells that had been transfected with *GJA1*, the gene encoding Cx43, were analyzed for Cx43 expression and functional gap junctions. We previously reported that H125 cells express negligible Cx43 or GJIC [12]. After transfection with *GJA1*, the cells (H125-CX43) exhibited a robust expression of the protein as shown by Western blot and the presence of punctate Cx43 immunofluroescence staining between adjacent cells (Figure 1A,D). The empty vector-transfected cells (H125-NEO) exhibited no Cx43 protein expression by Western blot or immunofluorescence. Cx26, Cx32, and Cx46, which are also expressed by lung epithelial cells [21], were not detected (Figure S1). When the cells were evaluated for functional gap junctions, the scrape-loading/dye transfer (SL/DT) assay revealed more GJIC in H125-Cx43 than H125-NEO cells (Figure 1B,C). Interestingly, the morphology of H125-CX43 cells was more cobblestone-like than H125-NEO cells (Figure 1B).

**Figure 2:**
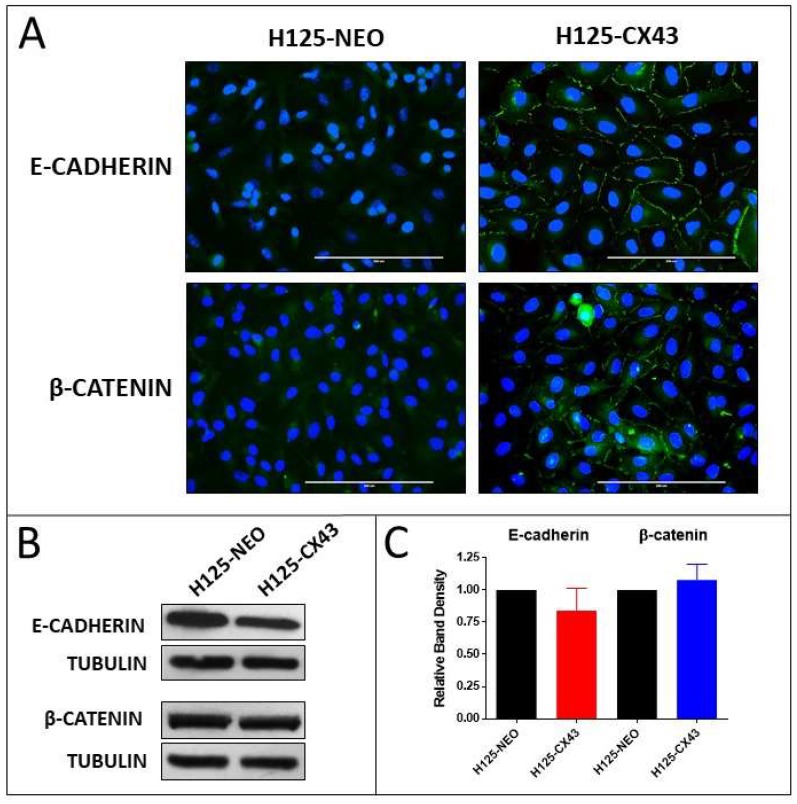
Localization and expression of E-cadherin and β-catenin in H125 cells. (**A**) Fluorescent FITC immunostaining of E-cadherin and β-catenin with DAPI staining of nuclei, scale bars: 200 µm; (**B**) Western blots of E-cadherin and β-catenin and (**C**) densitometric analysis of band densities normalized to tubulin loading control and to H125-NEO cells (no statistically significant differences; one-sample t-test, mean ± S.D., *n* = 3 replicate experiments).

Correspondingly, E-cadherin and β-catenin were more organized and localized around the periphery of H125-CX43 cells compared to diffuse cytoplasmic staining in H125-NEO cells (Figure 2A). Western blots indicated both cell lines expressed comparable amounts of the proteins (Figure 2B,C). These results indicate Cx43 is localized to the plasma membrane, forms functional gap junctions, and induces a more epithelial-like morphology when expressed in H125 cells. This suggests that a mesenchymal-to-epithelial (MET) change occurred in the Cx43-expressing cells, although additional studies are necessary to verify this.

### 2.2. Proliferation of the Transfected Cells

**Figure 3:**
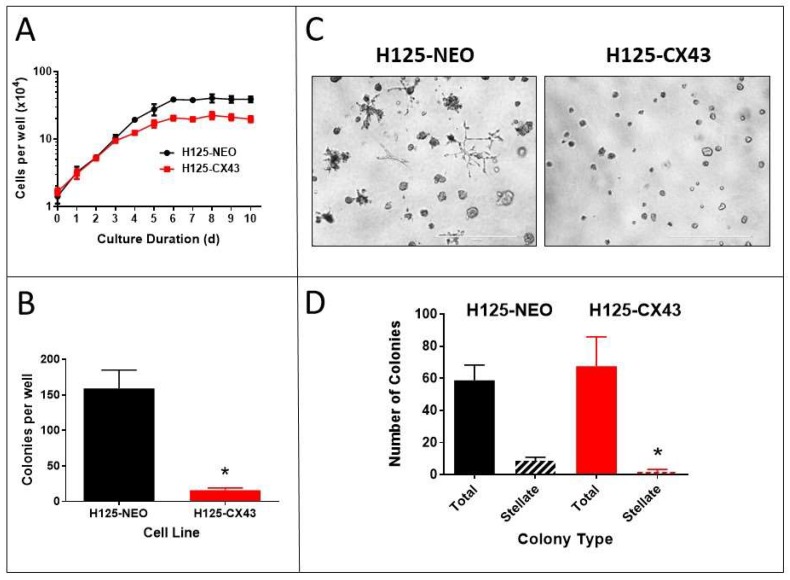
Connexin43 reduces the proliferation of H125 cells. (**A**) Growth of H125-NEO and H125-CX43 cells on plastic (mean ± S.D., *n* = 4 replicate experiments), (**B**) in soft agar, and (**C**) in Matrigel (scale bars: 1000 µm). (**D**) The number and types of colonies obtained after growth in Matrigel were enumerated. (**B**,**D**) * *p* < 0.01 compared to H125-NEO, Student’s *t*-test, mean ± S.D., *n* = 3 replicate experiments.

The proliferation of these cells on standard plastic tissue culture dishes was determined over 10 days (Figure 3A). The cells initially exhibited a similar rate of logarithmic growth over the first 3 days, but as culture density increased, H125-CX43 cell growth slowed and plateaued at an approximately 50% lower final density than H125-NEO cells. These data suggest Cx43 reduces proliferation when cells begin forming extensive contacts, but does not affect proliferation rates (doubling times) at lower density. This may be due to increased GJIC as cell density increases [22,23].

The ability of cells to grow in soft agar unattached to a solid substrate often correlates with neoplastic transformation [24]. H125-NEO cells formed numerous large colonies in soft agar whereas H125-Cx43 cells showed a much reduced capability (Figure 3B). This suggests Cx43 suppresses neoplastic transformation in these cells.

Neoplastic cells may also exhibit altered growth morphologies when cultured in an extracellular matrix compared to growth on plastic culture dishes. When H125-NEO and H125-CX43 cells were grown in culture medium that contained 0.5% Matrigel, numerous colonies of various size and shape arose (Figure 3C). There was no significant difference in the total number of colonies between the two cell types, but H125-CX43 cells generated fewer colonies with a stellate pattern of growth (Figure 3C,D).

### 2.3. Wound Closure and Invasion Assays

**Figure 4:**
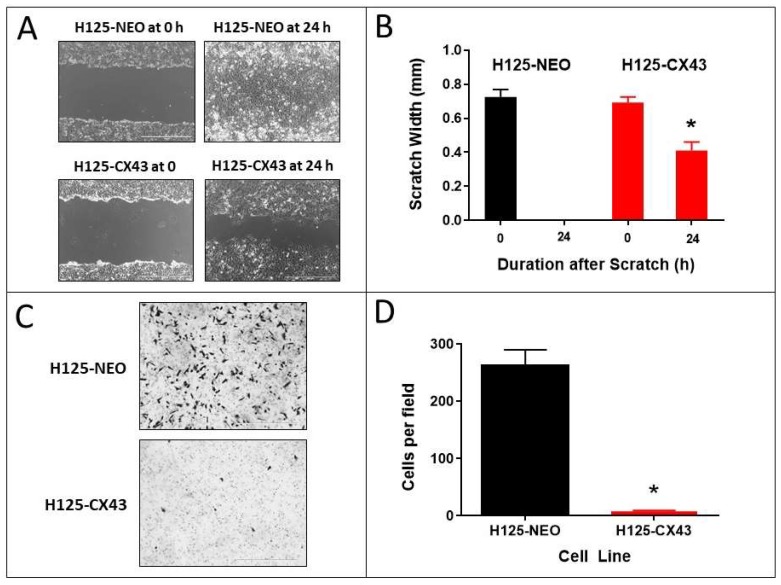
Connexin43 suppresses the migration and invasion of H125 cells. (**A**,**B**) Scratch assay of H125-NEO and H125-CX43 cells (scale bars: 1000 µm). (**C**,**D**) Matrigel transwell invasion with these cells (scale bars: 1000 µm). * *p* < 0.01 compared to H125-NEO, Student’s *t*-test, mean ± S.D., *n* = 3 replicate experiments.

The ability of cells to repair a scratch or wound in a monolayer culture over 24 h is predominantly due to the migratory capacity of the cells into the wound [25]. The H125-CX43 cells exhibited significantly decreased wound repair compared to H125-NEO cells. The latter completely repopulated the wound within 24 h whereas H125-CX43 cells covered only approximately 60% of the wound (Figure 4A,B).

Cell invasion through an extracellular matrix in vitro is suggestive of a high propensity for metastasis [25]. The H125 cell line was developed from a metastatic tumor in the skin [26] and, therefore, would be expected to be invasive in a matrix invasion assay. Accordingly, H125-NEO cells showed invasive ability through Matrigel, but this capacity was nearly absent in H125-CX43 cells (Figure 4C,D).

### 2.4. Cisplatin Sensitivity and Resistance

**Figure 5:**
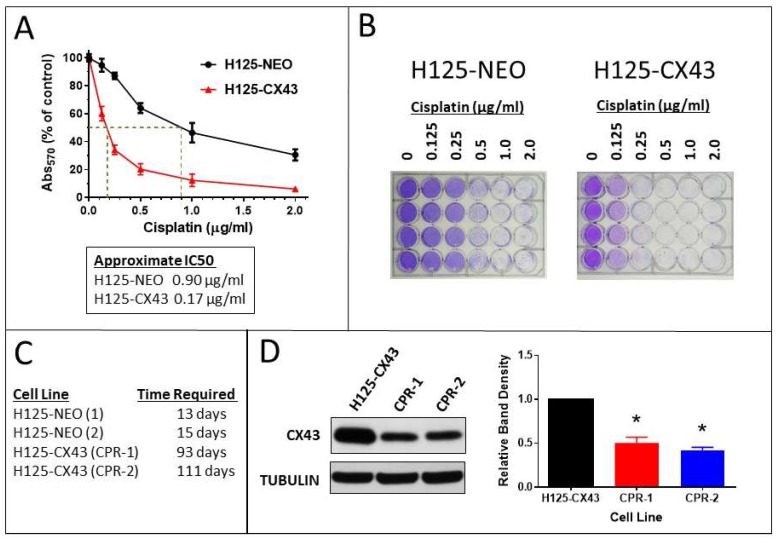
Connexin43-expressing H125 cells exhibit increased cytotoxicity and reduced ability to develop resistance to cisplatin. (**A**,**B**) The cisplatin concentration–response curves indicated approximately five-fold greater drug toxicity in H125-CX43 cells (mean ± S.D., *n* = 4 replicate experiments). (**C**) A longer duration of exposure was required to develop resistance to 2 µg/mL cisplatin in two separate trials with each cell line. (**D**) Western blot of Cx43 in cisplatin-naïve (H125-CX43) and resistant cells (CPR-1 and CPR-2); band densities were normalized to tubulin loading control and to H125-CX43 (* *p* < 0.01 compared to H125-CX43, one-sample *t*-test, mean ± S.D., *n* = 4 replicate experiments).

The expression of connexins and GJIC has been associated with increased sensitivity to cisplatin and other cytotoxic drugs, in some cases due to the bystander effect whereby toxic factors move between cells through gap junctions [27,28,29]. Accordingly, H125-CX43 cells were much more sensitive to cisplatin than H125-NEO cells (Figure 5A,B). The cisplatin IC_50_ values for the two cell lines were estimated to be 0.17 and 0.90 µg/mL, respectively, which is an approximate five-fold difference. In addition to this difference following one-time treatment, H125-NEO cells became resistant to cisplatin much more quickly during continuous exposure. In two separate trials, H125-NEO cells required 13 and 15 days to become resistant to 2 µg/mL cisplatin whereas H125-CX43 cells required 93 and 111 days (Figure 5C). Interestingly, H125-CX43 cisplatin-resistant cells from the two trials (CPR-1 and CPR-2) expressed less Cx43 than H125-CX43 non-resistant cells (Figure 5D). These data suggest Cx43 renders H125 cells more sensitive to cisplatin and less able to develop resistance.

### 2.5. Cancer Stem Cell Characteristics

**Figure 6:**
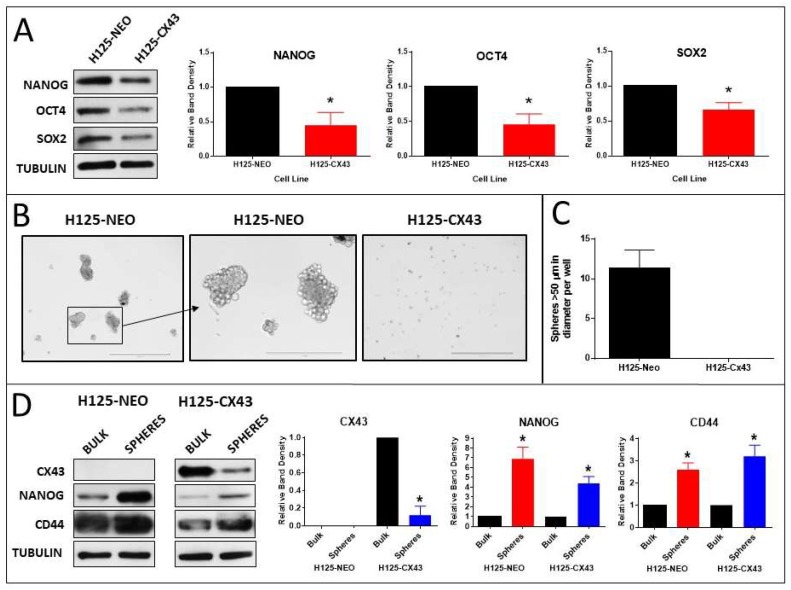
Connexin43 suppresses the expression of stem cell pluripotency markers and tumorsphere formation. (**A**) Western blots of stem cell pluripotency factors Nanog, Oct4, and Sox2 in H125-NEO and H125-CX43 cells; band densities were normalized to tubulin loading control and to H125-NEO (* *p* < 0.01 compared to H125-NEO, one-sample t-test, mean ± S.D., *n* = 3 replicate experiments); (**B**) images of tumorspheres after 3 weeks culture in stem cell medium; scale bars: 1000 µm (left and right panels) and 400 µm (middle panel); (**C**) quantification of spheres larger than 50 µm in diameter (mean ± S.D., *n* = 3 replicate experiments); (**D**) Western blots of Cx43, Nanog, and CD44 in bulk cells and spheres; band densities were normalized to tubulin loading control and to bulk cells (* *p* < 0.01 compared to bulk cells, one-sample *t*-test, mean ± S.D., *n* = 3 replicate experiments).

The effect of Cx43 on the expression of the CSC pluripotency markers Nanog, Oct4, and Sox2 was determined in the H125 cells. The content of all three markers was lower in H125-CX43 cells than in H125-NEO cells (Figure 6A). This suggested Cx43 decreased CSC abundance and/or expression of pluripotency markers in H125 cells.

To further address this, tumorsphere formation in the two lines was compared [30]. After 3 weeks of culture in sphere-forming medium, numerous large (>50 µm diameter) spheres developed in H125-NEO cultures, but none were evident in H125-CX43 cells; only small clusters of cells were present in the latter (Figure 6B,C). These small cell clusters were analyzed for Cx43 content by Western blot. Interestingly, the content of Cx43 was reduced in the small spheres compared to bulk cells (Figure 6D). Tumorspheres from H125-NEO cells (Figure 6B) did not exhibit Cx43 expression by Western blot (Figure 6D). Since tumorspheres may originate from CSCs [30], these results suggest Cx43 reduces the abundance of CSCs in H125 cells.

**Figure 7:**
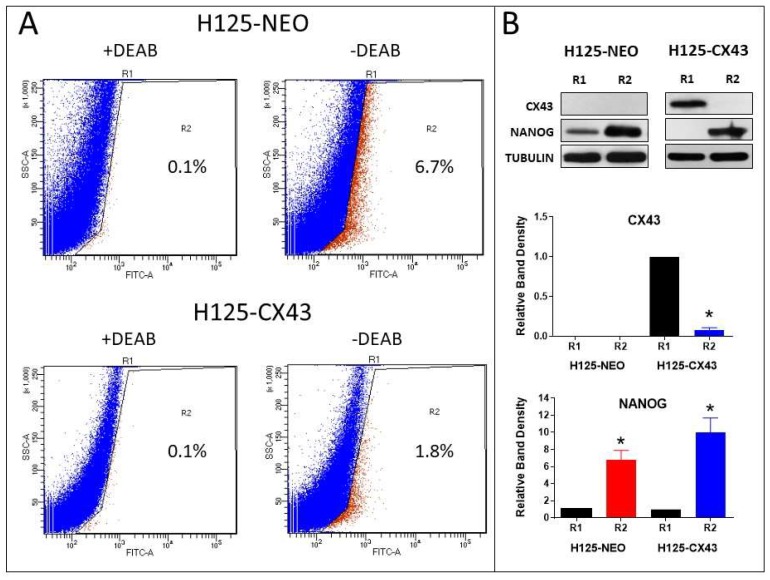
Connexin43 reduces the population of aldehyde dehydrogenase (ALDH)-positive H125 cells. (**A**) Dot plots of fluorescence assisted cell sorting (FACS)-sorted ALDH-low (R1 pool) and ALDH-high (R2 pool) with and without DEAB in H125-NEO and H125-CX43 cells; the percentages of R2 cells are shown on the plots. (**B**) Western blots of Cx43 and Nanog in R1 (ALDH-low) and R2 (ALDH-high) populations of H125-NEO and H125-CX43 cells; band densities were normalized to tubulin loading control and to R1 cells (* *p* < 0.01 compared to R1 cells, one-sample *t*-test, mean ± S.D., *n* = 3 replicate experiments).

To further address this, ALDH expression, which is a marker of lung CSCs [31], was used to isolate populations of CSC and non-CSC cells in the H125 cells. The percentage of ALDH-high (R2 population) is shown for each cell type (Figure 7A). Western blots indicated Cx43 content was lower in the R2 pool of H125-CX43 cells compared to the R1 population; Cx43 was not detected in R1 or R2 cells of H125-NEO (Figure 7B). Greater amounts of Nanog were present in the R2 populations of both cell types which also suggests these are CSC-like cells (Figure 7B). These data are similar to the tumorsphere results above; they suggest enhanced Cx43 expression reduces CSC abundance and residual CSCs have less Cx43 expression than bulk cells.

### 2.6. Tumorsphere Formation and Stem Cell Marker Expression in H522 and H661 Cells

**Figure 8:**
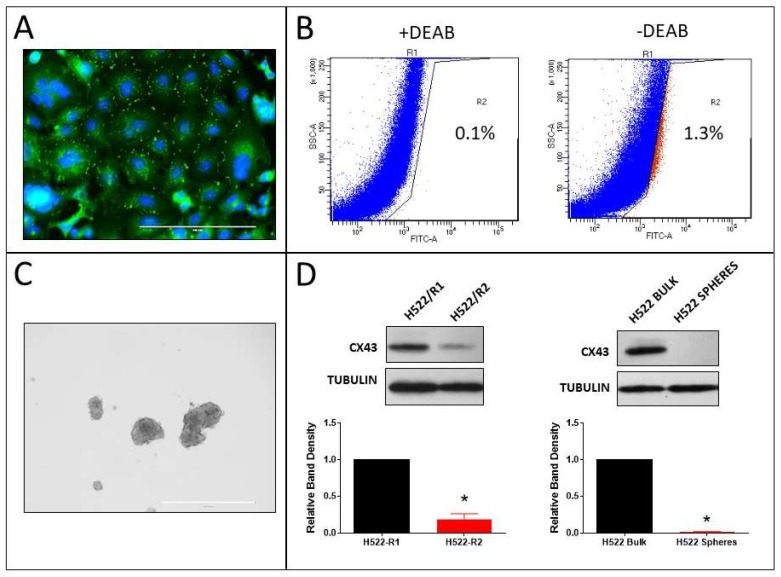
Expression of Cx43 in ALDH-positive cells and tumorspheres derived from H522 cells. (**A**) Cx43 immunostaining (scale bar: 200 µm); (**B**) dot plots of FACS-sorted ALDH-low (R1 pool) and ALDH-high (R2 pool) with and without DEAB (the percentages of R2 cells are shown on the plots); (**C**) H522 tumorspheres (scale bar: 1000 µm); and (**D**) Western blots of Cx43 in R1 (ALDH-low) and R2 (ALDH-high) populations (left blots) and bulk cells and tumorspheres (right blots); Cx43 band densities were normalized to tubulin loading control and to R1 or bulk cells (* *p* < 0.01 compared to R1 or bulk cells, one-sample t-test, mean ± S.D., *n* = 3 replicate experiments).

Our previous study showed that the human non-small cell lung cancer (NSCLC) cell lines H522 (adenocarcinoma) and H661 (large cell carcinoma) exhibit Cx43 expression and GJIC endogenously [12]. Immunostaining of Cx43 in H522 cells confirmed this (Figure 8A). ALDH-positive cells were sorted (Figure 8B), tumorspheres (Figure 8C) were generated from these cells, and then Cx43 content was compared between the R1 and R2 populations and the bulk cells and tumorspheres (Figure 8D). Accordingly, the ALDH-positive R2 population and tumorspheres had lower Cx43 content.

**Figure 9:**
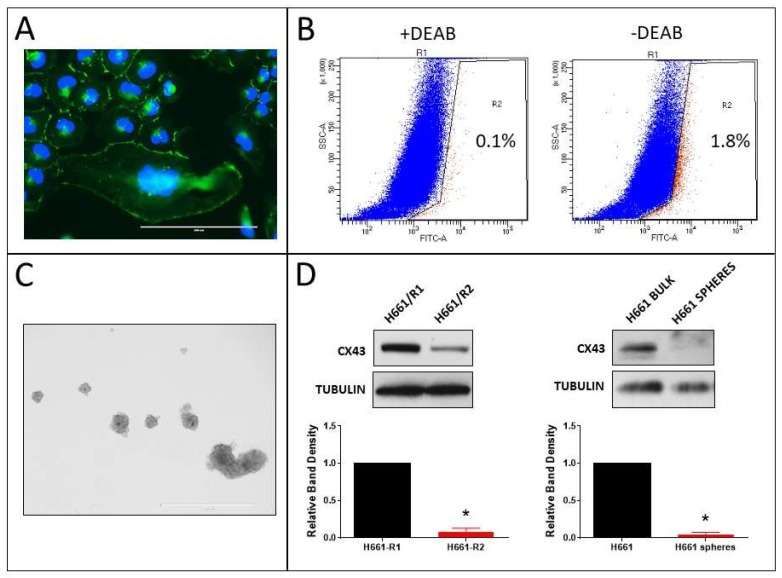
Expression of Cx43 in ALDH-positive cells and tumorspheres derived from H661 cells. (**A**) Cx43 immunostaining (scale bar: 200 µm); (**B**) dot plots of FACS-sorted ALDH-low (R1 pool) and ALDH-high (R2 pool) with and without DEAB (the percentages of R2 cells are shown on the plots); (**C**) H661 tumorspheres (scale bar: 1000 µm); and (**D**) Western blots of Cx43 in R1 (ALDH-low) and R2 (ALDH-high) populations (left blots), and bulk cells and tumorspheres (right blots); Cx43 band densities were normalized to tubulin loading control and to R1 or bulk cells (* *p* < 0.01 compared to R1 or bulk cells, one-sample t-test, mean ± S.D., *n* = 3 replicate experiments).

Similar results were observed in H661 cells. Cx43 was endogenously expressed (Figure 9A) and Cx43 content was lower in ALDH-positive R2 cells and tumorspheres (Figure 9B–D). The results indicate that even in NSCLC cell lines with endogenous Cx43 expression, CSC-like cells are present and they have lower expression of the protein than bulk cells.

## 3. Discussion

NCI-H125 cells were derived from a cutaneous metastasis of a human lung adenocarcinoma [26]. Here we show that the expression of Cx43 in these highly malignant cells dramatically normalized their phenotype and reduced the number of CSCs and CSC markers. The H125-CX43 cells acquired an epithelial, cobblestone-like morphology with organized E-cadherin and β-catenin at the plasma membrane, and exhibited contact inhibition of growth at a lower cell density than H125-NEO cells. The H125-CX43 cells were less able to grow in soft agar, repair a scratch wound, and invade through Matrigel. They were also more sensitive to cisplatin and took longer to develop resistance. Resistant cells expressed less Cx43 than cells not exposed to the drug.

These data corroborate previous reports of the phenotype-normalizing effects of Cx43 in lung cancer cells. We first reported that Cx43 transfection of highly malignant murine lung carcinoma cells dramatically reduced their growth in vitro and tumorigenicity and was associated with increased expression of the cyclin-CDK inhibitor p27 and decreased cyclin D1 [16,32]. Other groups have made similar observations in Cx43-transfected rodent and human lung cancer cell lines [17,19]. Accordingly, the expression of Cx43 in human NSCLC was lowest in highly malignant tumors and correlated with decreased survival [14,27,33]. The knockout of *Gja1* rendered mice more susceptible to chemical induction of lung tumors [34,35].

As noted in the Introduction, a limited number of studies of GJIC and connexin expression in CSCs have been undertaken and the results are not consistent. Some studies indicate connexins suppress CSCs, whereas others reveal connexins drive self-renewal of CSCs, and in some cases, the connexin expressed is a novel member not seen in the non-CSCs [5,6,7,8,9,10,11]. Regarding lung cancer, higher levels of Cx43 and GJIC were seen in A549 lung cancer stem cells compared to bulk cells, and this was suggested as a possible drug target against brain metastasis [9].

In the present study, Cx43 reduced the expression of lung CSC markers and CSC abundance in agreement with some of these previous studies [6,11]. The H125-CX43 cells exhibited less Nanog, Oct4, and Sox2, three markers of pluripotency that are necessary for stem cell maintenance and associated with lung CSCs [36]. The cells also exhibited reduced tumorsphere formation and fewer ALDH high-expressing cells. The small tumorspheres that did arise and ALDH high-expressing cells that were present in the H125-CX43 cells had reduced Cx43 content compared to bulk H125-CX43 cells. Similarly, in H522 and H661 cells, which endogenously express Cx43, the expression of the protein in tumorspheres and ALDH high-expressing cells was much lower than in bulk cells. Other connexins that are expressed by lung epithelial cells (Cx26, Cx32, and Cx46) [21] were not detected in H125-NEO and H125-CX43 cells or tumorspheres derived from them. These data suggest Cx43 expression is reduced in lung CSCs; its upregulation can inhibit neoplasia by suppressing lung CSC abundance in addition to reversing the cancer phenotype, and expression of a novel connexin does not drive these cells.

The mechanism of CSC suppression and phenotypic normalization by Cx43 may involve GJIC between CSCs that ectopically express Cx43 and the much more populous non-CSC bulk cells. If CSCs form gap junctions with bulk cells, molecules that regulate stemness and that are over- or underexpressed in CSCs could be buffered by the larger bulk population [37,38,39]. This buffering of stem cell regulatory signals by GJIC would likely need to persist for a sufficient time that re-programming of the CSCs occurred. If GJIC was not sustained, CSCs did not express ectopic *GJA1*, or they were incapable of forming gap junctions (because of poor cell–cell adhesion or other mechanisms); junctional coupling and signal buffering would not occur; and CSCs would maintain their stemlike characteristics. 

What might those gap junction-permeable signals be? That is a difficult question to address experimentally since there are tens of thousands of cellular molecules and ions small enough to pass through gap junction pores. These include second messengers such as cyclic nucleotides and Ca^+2^, metabolites and other ions, and miRNAs and small peptides. Several miRNAs such as miR-99a and miR-410 are aberrantly expressed in lung cancer stem cells and play roles in drug resistance, metastasis, and stemness [37,40,41] and might be buffered by GJIC. 

Other mechanisms might not involve GJIC. The cytoplasmic domains of many connexins including Cx43 interact with signaling and structural/cytoskeletal proteins and this can impact adhesion, proliferation, invasion, and other functions in a GJIC-independent manner [42]. Connexin43 may interact with proteins that control critical lung CSC signaling pathways or gene expression independent of gap junctions. Related to this, increased localization of E-cadherin and β-catenin to the plasma membrane and enhanced cobblestone morphology was seen in H125-CX43 cells which suggested that Cx43 induced MET. An increase in adherens junction protein assembly at the plasma membrane may reduce the transcriptional activity of junctional proteins that drive stemness [43]. In support of this, Yu et al. previously reported that A549 lung adenocarcinoma cells that were resistant to cisplatin had acquired an EMT phenotype and ectopic expression of CX43 reversed this morphological change and drug resistance [44]. Other groups also reported ectopic expression of Cx43-reversed EMT in breast cancer cells [45] or that retinoid-induced EMT in colon cancer cells was associated with increased Cx43 expression [46].

Lastly, putative degradation products of Cx43 as well as truncated forms of the protein that are encoded from internal AUG start codons have been identified [47,48,49]. These may localize to the nucleus and affect gene expression and cellular functions [50,51]. Salat-Canela et al. reported that the internal translation of a 20 kDa form of Cx43 occurs in A549 and HOP-62 lung cancer and other cancer cells, and this is under the control of the Mnk1/2 pathway [52]. Although we did not observe Cx43 immunostaining in the nuclei of H125-CX43 cells or truncated forms of the protein on Western blots, undetectable amounts might be present to suppress CSCs. 

Cancer stem cells are typically more resistant to cytotoxic drugs and radiotherapy than bulk cells and often develop resistance [1,2]. Gap junctions facilitate bystander toxicity whereby neighboring cells can be killed via gap junction-mediated passage of cytotoxic molecules from damaged cells to neighbors [28]. This is true with cisplatin-targeted lung cancer cells as we recently reported [27]. The present study demonstrated that Cx43-deficient cells were less sensitive to cisplatin and more easily developed resistance to the drug than Cx43-expressing cells. This suggests that Cx43 upregulation in lung CSCs could increase drug sensitivity and decrease resistance via direct cell killing and bystander toxicity.

This study focused on established human lung cancer cell lines. Further study is necessary to determine if CSCs present in clinical cases of lung cancer are also deficient in gap junctions and connexin expression. The decreased expression of Cx43 in lung cancer has been attributed to epigenetic silencing of *GJA1* [13], but whether this is true in lung CSCs is not known. Additionally, *GJA1* mutations are frequent in some hypermutated lung cancers [27]. These mutations might also impact transcriptional control and/or the functional motifs of Cx43 that regulate its stability, localization, gap junction assembly, and protein–protein interactions. Identification of the mechanism(s) of Cx43 suppression in lung CSCs and the development of methods to enhance its expression and GJIC may improve lung cancer treatment and survival.

## 4. Materials and Methods

### 4.1. Cells and Culture Conditions

The human NSCLC cell lines, NCI-H125 (adenocarcinoma), NCI-H522 (adenocarcinoma), and NCI-H661 (large cell carcinoma), were purchased from the American Type Culture Collection (Rockville, MD, USA). All lines were maintained in complete medium that consisted of RPMI1640 medium supplemented with fetal bovine serum (10% v/v) and gentamicin (50 µg/mL) (Fisher Scientific, Chicago, IL, USA). The cells were cultured in standard plastic tissue culture flasks and dishes and were passaged by trypsinization.

### 4.2. Transfection of Cells, SL/DT Assay, and Detection of Cx43

The H125 cells were transfected with pcDNA3.1-CX43, a Cx43 expression vector that contains *GJA1* under the transcriptional control of the human cytomegalovirus promoter [53]. Control cells were transfected with the empty vector (pcDNA3.1). Stable transfectants were selected by culture in a medium supplemented with G418 (0.4 mg/mL; Fisher Scientific, Chicago, IL, USA) over five passages.

Transfected cells were evaluated for functional gap junctions by scrape-loading/dye transfer (SL/DT) assay, gap junction formation by immunofluorescence staining, and Cx43 content by Western blotting. Briefly, for the SL/DT assay [54], monolayers of cells in 35-mm dishes were washed with serum-free medium, then covered in 1 mL of the same medium that contained 0.5% Lucifer Yellow CH (Fisher Scientific), and then three scrapes were made across the monolayer with a pipet tip. This allowed the dye to enter cells along the scrape edge. After 5 min of incubation to allow the entrapped dye to diffuse through gap junctions into neighboring cells, the cultures were washed with serum-free medium, fixed with 10% buffered formalin, and observed and photographed with an EVOS FL epifluoresence microscope (Fisher Scientific, Chicago, IL, USA). At 10 points selected randomly along the scrape, the number of cells perpendicular to the scrape that contained fluorescent dye was determined. The mean of these measurements was considered the SL/DT index for that culture.

For immunofluorescent staining of Cx43, E-cadherin, or β-catenin, cells were grown on sterile glass coverslips and then fixed with ice-cold 10% acetic acid in methanol when nearly confluent. Cells were then washed with PBS, incubated with normal goat serum (005-000-001, Jackson Immunoresearch Laboratories, West Grove, PA, USA) at 1:100 in PBS for 1 h, primary antibody (anti-Cx43 (610062) or anti-E-cadherin (610181) mouse monoclonal antibody from BD Biosciences (San Jose, CA, USA) or anti-β-catenin (sc-7963) from Santa Cruz Biotechnology (Santa Cruz, CA, USA)) at 1:100 in PBS for 4 h, and finally FITC-conjugated goat anti-mouse IgG antibody (115-095-071, Jackson Immunoresearch Laboratories) at 1:100 in PBS for 2 h. Coverslips were mounted with DAPI Fluoromount-G (0100-20, Southern Biotech, Birmingham, AL, USA) at room temperature. Images of stained cells were obtained with an EVOS FL epifluorescence microscope.

For Western blotting of Cx43 and other proteins, cells were lysed in ice-cold RIPA buffer (Fisher Scientific) supplemented with phenylmethylsulfonyl fluoride and protease inhibitor cocktail (Sigma-Aldrich, ST. Louis, MO, USA) followed by sonication on ice and centrifugation at 13,000× *g* for 15 min to remove debris. Protein concentration in the supernatant was determined with the BioRad detergent-compatible (DC) protein assay (BioRad Corporation, Hercules, CA, USA). Pre-cast 10% polyacrylamide SDS-PAGE gels (Fisher Scientific) were loaded with 5–40 µg of protein per lane depending on the experiment. After electrophoresis and transfer to the Immobilon membrane (Sigma-Aldrich), proteins were detected using primary antibodies: anti-Cx43 (610062) and anti-E-cadherin (610181) from BD Biosciences; anti-Oct4A (C30A3), anti-Nanog (D73G4), anti-Sox2 (D6D9), and anti-CD44 (156-3C11) from Cell Signaling Technology (Danvers, MA, USA); and anti-β-catenin (sc-7963), anti-Cx26 (sc-293223), anti-Cx32 (sc-59948), anti-Cx46 (sc-365394), and anti-α-tubulin (sc-8035) from Santa Cruz Biotechnology (Santa Cruz, CA, USA). Secondary antibodies were horseradish peroxidase (HRP)-conjugated donkey anti-rabbit IgG (711-035-152, Jackson Immunoresearch Laboratories) and HRP-conjugated goat anti-mouse IgG (554002, BD Biosciences). Band densities were quantified using public domain Image J software (Version 1.52a, http://imagej.nih.gov/ij, National Institutes of Health, Bethesda, MD, USA).

### 4.3. Cell Proliferation

Cell proliferation was determined by plating 2 × 10^4^ cells per well of 24-well plates in complete medium and then trypsinizing and counting the cells daily over the next 10 days. Cells were refed every other day and counted manually with a hemocytometer after trypsinization.

### 4.4. Growth in Soft Agar

The ability of cells to grow in soft agar was determined. The bottoms of 60-mm dishes were coated with 1 mL of 0.5% low melting point agarose (Fisher Scientific) in complete culture medium, then 2 × 10^3^ cells suspended in 1 mL of 0.3% agarose in complete medium were plated over this base layer. One milliliter of complete culture medium was added over this top layer, and 2 weeks later, the cells were fixed with 10% buffered formalin (Fisher Scientific). Colonies at least 0.5 mm in diameter were counted manually.

### 4.5. Colony Formation in Matrigel

The growth of cells in phenol-red free Matrigel (DLW356237, Sigma-Aldrich) was analyzed. The cells (2.5 × 10^3^ per well) were suspended in 1 mL of 0.5% Matrigel in complete culture medium and plated on top of a 100% Matrigel base layer (0.5 mL) in 24-well plates. After culture for 1 week, colonies were counted and photographed.

### 4.6. Wound Repair/Scratch Assay

The ability of cells in a confluent monolayer to migrate and repair a scratch “wound” was determined. A scratch was first made across the monolayer (0 h time point) using a 200 µL plastic pipet tip, then the scratch was photographed immediately at a time of 0 h and 24 h later. The width of the scratch at 0 h and 24 h was measured at 10 points selected randomly along the scratch.

### 4.7. Matrigel Invasion Assay

The invasiveness of cells through an extracellular matrix was determined using a Matrigel-coated membrane culture dish inserts (354483, Fisher Scientific). Cells (2.5 × 10^4^ in 0.5 mL of serum-free medium) were plated into inserts and these were placed into wells of 24-well plates that contained 0.75 mL of serum-containing medium that acted as a chemoattractant. After culture for 24 h, invasion of cells through the matrix and across the membrane was determined. Noninvasive cells on top of the membrane were first removed with a cotton swab and then invasive cells on the underside were fixed with 10% buffered formalin, stained with crystal violet, and counted.

### 4.8. Drug Cytotoxicity and Resistance

To determine the cytotoxicity of cisplatin, cells (5 × 10^4^ per well) were plated into 24-well plates and treated the next day with cisplatin (232120, Calbiochem, San Diego, CA, USA) which was first dissolved in sterile water. Control cultures were treated with sterile water. Two days later, the cultures were washed and refed with complete medium, cultured an additional 3 days, and then fixed with 10% buffered formalin. Residual cells were then stained with 2% crystal violet, washed with water, air-dried, and dissolved in 2% SDS (1 mL/well). The absorbance at 570 nm of the solution was measured with a spectrophotometer.

The time needed for cells to become resistant to cisplatin was also determined. The cells were first incubated with a low concentration of cisplatin that caused approximately 25% cell death. When cells reached confluence, they were trypsinized and replated at approximately one-third confluency and re-treated with cisplatin at twice the previous concentration. This process continued until the cells were able to successfully grow to full confluence after one passage at a drug concentration of 2 µg/mL. The number of days required to reach this threshold was recorded.

### 4.9. Tumorsphere Formation

The sphere-forming ability of cells was determined by culture in stem cell medium consisting of DMEM/F12 medium (SH30023.01) supplemented with 2% v/v B27 (17504044), 20 ng/mL EGF (PHG0311), 20 ng/mL bFGF (PHG0264), 50 µg/mL gentamicin (17-5187), and 0.4 mg/mL G418 (30-234-C1), all purchased from Fisher Scientific. One-thousand cells in 3 mL of stem cell medium were plated per well into 6-well plates and cultured for 3 weeks. Every 3 days, fresh growth factors were added to each well. At the end of the culture period, spheres larger than 50 µm were counted. Spheres were collected by centrifugation and lysed in radioimmunoprecipitation assay (RIPA) buffer (Fisher Scientific, Chicago, IL, USA) for Western blot studies.

### 4.10. Isolation of ALDH-Positive Cells

Lung CSCs often express higher levels of aldehyde dehydrogenase (ALDH) than non-CSCs, and this property can be used to isolate CSCs [30]. The ALDEFLUOR Stem Cell identification Kit (01700, STEMCELL Technologies, Cambridge, MA, USA) was used to identify cells that expressed high levels of ALDH in the lung cancer cell lines. Briefly, 1 × 10^6^ suspended cells were incubated with or without the ALDH inhibitor, N,N-diethylaminobenzaldehyde (DEAB), in ALD Buffer for 45 min at 37 °C with occasional mixing. Then the cells were pelleted, washed, resuspended in ice-cold ALD buffer supplied with the kit, and sorted by fluorescence assisted cell sorting (FACS). The ALDH high-expressing cells (R2 pool) were identified by gating out low-expressing cells (R1 pool). The R1 and R2 cells were collected and used for Western blotting.

## 5. Conclusions

The results presented in this paper demonstrate for the first time that the gap junction protein, Cx43, when expressed in human metastatic lung carcinoma cells, suppresses several phenotypic characteristics of lung CSCs, including resistance to cisplatin, and reduces their abundance amongst the bulk cancer cell population. This suggests that upregulation of this protein in lung CSCs may improve lung cancer therapy and survival.

## Supplementary Materials

The following are available online at http://www.mdpi.com/2072-6694/11/2/175/s1, Figure S1: Western blots of Cx26, Cx32, and Cx46 in H125-NEO and H125-CX43 cells and tumorspheres derived from them.
