# Supplementary material for: Connexin43 Suppresses Lung Cancer Stem Cells
Source: Cancers (Basel). 2019 Feb 2;11(2):175. doi: 10.3390/cancers11020175 (PMC6406368; doi:10.3390/cancers11020175)
Supplement: Supplementary file 1 [file cancers-11-00175-s001.pdf]

Supplementary Materials: Connexin43 Suppresses Lung Cancer Stem Cells

Randall J. Ruch

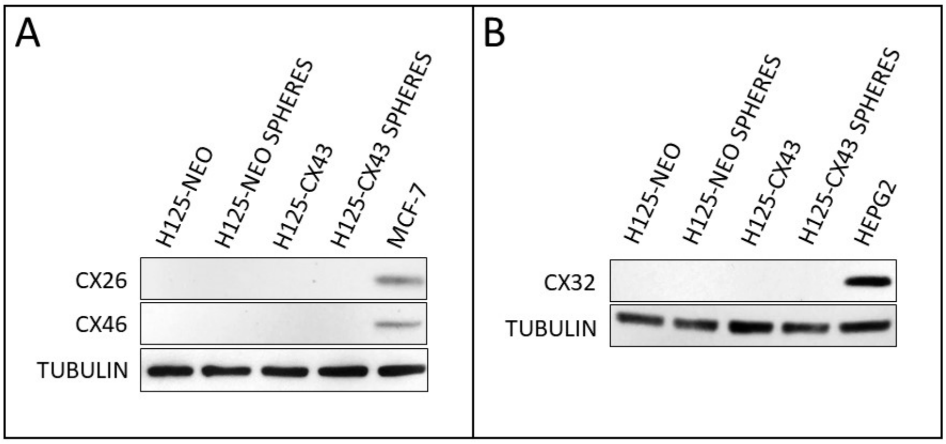

Figure S1. Western blots of Cx26, Cx32, and Cx46 in H125-NEO and H125-CX43 cells and tumorspheres derived from them. MCF-7 cells were used as a positive control for Cx26 and Cx46 and HepG2 cells were used for Cx32.

© 2019 by the authors. Licensee MDPI, Basel, Switzerland. This article is an open access article distributed under the terms and conditions of the Creative Commons Attribution (CC BY) license (http://creativecommons.org/licenses/by/4.0/).
